# Cyclooxygenase-2 up-regulates vascular endothelial growth factor via a protein kinase C pathway in non-small cell lung cancer

**DOI:** 10.1186/1756-9966-30-6

**Published:** 2011-01-10

**Authors:** Honghe Luo, Zhenguang Chen, Hui Jin, Mei Zhuang, Tao Wang, Chunhua Su, Yiyan Lei, Jianyong Zou, Beilong Zhong

**Affiliations:** 1 Department of Thoracic Surgery, The First Affiliated Hospital, Sun Yat-sen University, Guangzhou (510080), Guangdong, People's Republic of China; 2 Private Medical Center, The First Affiliated Hospital, Sun Yat-sen University, Guangzhou (510080), Guangdong, People's Republic of China; 3 Center for Stem Cell Biology and Tissue Engineering, Sun Yat-sen University, Key Laboratory for Stem Cells and Tissue Engineering, Ministry of Education, Guangdong, People's Republic of China; 4 Department of Thoracic Surgery, The Fifth Affiliated Hospital, Sun Yat-sen University, Zhuhai (519000), Guangdong, People's Republic of China

## Abstract

**Background:**

Vascular endothelial growth factor (VEGF) expression is up-regulated via a cyclooxygenase-2 (COX-2)-dependent mechanism in non-small cell lung cancer (NSCLC), but the specific signaling pathway involved is unclear. Our aim was to investigate the signaling pathway that links COX-2 with VEGF up-regulation in NSCLC.

**Material and methods:**

COX-2 expression in NSCLC samples was detected immunohistochemically, and its association with VEGF, microvessel density (MVD), and other clinicopathological characteristics was determined. The effect of COX-2 treatment on the proliferation of NSCLC cells (A549, H460 and A431 cell lines) was assessed using the tetrazolium-based MTT method, and VEGF expression in tumor cells was evaluated by flow cytometry. COX-2-induced VEGF expression in tumor cells was monitored after treatment with inhibitors of protein kinase C (PKC), PKA, prostaglandin E2 (PGE_2_), and an activator of PKC.

**Results:**

COX-2 over-expression correlated with MVD (*P *= 0.036) and VEGF expression (*P *= 0.001) in NSCLC samples, and multivariate analysis demonstrated an association of VEGF with COX-2 expression (*P *= 0.001). Exogenously applied COX-2 stimulated the growth of NSCLCs, exhibiting EC_50 _values of 8.95 × 10^-3^, 11.20 × 10^-3^, and 11.20 × 10^-3 ^μM in A549, H460, and A431 cells, respectively; COX-2 treatment also enhanced tumor-associated VEGF expression with similar potency. Inhibitors of PKC and PGE_2 _attenuated COX-2-induced VEGF expression in NLCSCs, whereas a PKC activator exerted a potentiating effect.

**Conclusion:**

COX-2 may contribute to VEGF expression in NSCLC. PKC and downstream signaling through prostaglandin may be involved in these COX-2 actions.

## Background

Cyclooxygenase-1 and -2 (COX-1 and COX-2) are the rate-limiting enzymes for the synthesis of prostaglandins from arachidonic acid [1]. These two isoforms play different roles, with COX-2 in particular suggested to contribute to the progression of solid tumors [2]. Generally, constitutive activation of COX-2 has been demonstrated in various tumors of the lung, including atypical adenomatous hyperplasia [3], adenocarcinoma [4], squamous cell carcinoma [5] and bronchiolar alveolar carcinoma [6], and its over-expression has been associated with poor prognosis and short survival of lung cancer patients [7]. However, although altered COX-2 activity is associated with malignant progression in non-small cell lung cancer (NSCLC), the intrinsic linkage has remained unclear. COX-2 is believed to stimulate proliferation in lung cancer cells via COX-2-derived prostaglandin E2 (PGE_2_) and to prevent anticancer drug-induced apoptosis [8]. COX-2 has also been suggested to act as an angiogenic stimulator that may increase the production of angiogenic factors and enhance the migration of endothelial cells in tumor tissue [9]. Interestingly, COX-2 levels are significantly higher in adenocarcinoma than in squamous cell carcinoma, an observation that is difficult to account for based on the findings noted above [10].

More importantly, recent evidence has demonstrated that COX-2-transfected cells exhibit enhanced expression of VEGF [11], and COX-2-derived PGE_2 _has been found to promote angiogenesis [12]. These results suggest that up-regulation of VEGF in lung cancer by COX-2 is dependent on downstream metabolites rather than on the level of COX-2 protein itself. Although thromboxane A2 had been identified as a potential mediator of COX-2-dependent angiogenesis [13], little is known about the specific downstream signaling pathways by which COX-2 up-regulates VEGF in NSCLC.

Here, on the basis of the association of COX-2 expression with VEGF in both NSCLC tumor tissues and cell lines, we treated NSCLC cells with concentrations of COX-2 sufficient to up-regulate VEGF expression and evaluated the signaling pathways that linked COX-2 stimulation with VEGF up-regulation.

## Material and methods

### Patients and specimens

In our study, tissues from 84 cases of NSCLC, including adjacent normal tissues (within 1-2 cm of the tumor edge), were selected from our tissue database. Patients had been treated in the Department of Thoracic Surgery of the First Affiliated Hospital of Sun Yat-sen University from May 2003 to January 2004. None of the patients had received neoadjuvant chemotherapy or radiochemotherapy. Clinical information was obtained by reviewing the preoperative and perioperative medical records, or through telephone or written correspondence. Cases were staged based on the tumor-node-metastases (TNM) classification of the International Union Against Cancer revised in 2002 [14]. The study has been approved by the hospital ethics committee. Patient clinical characteristics are shown in Table 1. Paraffin specimens of these cases were collected, and 5-mm-thick tissue sections were cut and fixed onto siliconized slides. The histopathology of each sample was studied using hematoxylin and eosin (H&E) staining, and histological typing was determined according to the World Health Organization (WHO) classification [15]. Tumor size and metastatic lymph node number and locations were obtained from pathology reports.

**Table 1:** Association of COX-2 expression in NSCLC with clinical and pathologic factors (*χ*^2 ^test)

	Total	COX-2 low expression *n *(%)	COX-2 high expression *n *(%)	*P*
**Sex**				
**Male**	63	33 (52.4)	30 (47.6)	0.803
**Female**	21	12 (57.1)	9 (42.9)	
**Age**				
**≤60 years**	44	23 (52.3)	21 (47.7)	0.830
**> 60 years**	40	22 (55.0)	18 (45.0)	
**Smoking**				
**Yes**	38	21 (55.3)	17 (44.7)	0.828
**No**	46	24 (52.2)	22 (47.8)	
**Differentiation**				
**Well and moderate**	40	20 (50.0)	20 (50.0)	0.662
**Poor**	44	25 (56.8)	19 (43.2)	
**TNM stage**				
**I**	44	21 (47.7)	23 (52.3)	0.357
**II**	19	10 (52.6)	9 (47.4)	
**III + IV**	21	14 (66.7)	7 (33.3)	
**Histology**				
**Adeno**	34	18 (52.9)	16 (47.1)	0.561
**SCC**	45	23 (51.1)	22 (48.9)	
**Large cell carcinoma**	5	4 (80.0)	1 (20.0)	
**VEGF expression**				
**High**	42	12 (28.6)	30 (71.4)	0.000
**Low**	42	33 (78.6)	9 (21.4)	
**MVD expression**				
**High**	28	10 (35.7)	18 (64.3)	0.036
**Low**	56	35 (62.5)	21 (37.5)	

Abbreviations: Adeno, adenocarcinoma; SCC, squamous cell carcinoma.

### Cell culture and experimental agents

The NSCLC lines used in this experiment (A549, H460, and A431) were obtained from the American Type Culture Collection; human bronchial epithelial cells (HBE) were used as controls. A549 cells were cultured in 80% Roswell Park Memorial Institute (RPMI) 1640 medium supplemented with 20% fetal bovine serum (FBS); H460, A431, and HBE cells were cultured in 90% Dulbecco's Modified Eagle medium (DMEM) supplemented with 10% FBS. Cells were maintained at 37°C in a humidified 5% CO_2 _atmosphere. As cells approached confluence, they were split following treatment with Trypsin-EDTA; cells were used after four passages. COX-2, methylthiazolyl tetrazolium (MTT), the PGE_2 _receptor (EP1/2) antagonist AH6809 (catalog number 14050), and selective inhibitors of PKA (KT5720, catalog number K3761), and PKC (RO-31-8425) were all purchased from Sigma-Aldrich Co., Ltd (St. Louis, MO, USA). An antibody against human COX-2 was obtained from Invitrogen Biotechnology (catalog number COX 229, Camarillo, CA, USA), antibody against human VEGF was obtained from Santa Cruz Biotechnology (catalog number C-1, Santa Cruz, CA, USA), and antibody against human CD34 was obtained from Lab Vision (catalog number MS-363, Fremont, CA, USA). The selective PKA activator phorbol myristate acetate (PMA) was purchased from Promega (Madison, WI, USA).

### Immunohistochemical staining and assessment of COX-2, VEGF, and MVD

Immunohistochemical staining was carried out using the streptavidin-peroxidase method. Briefly, each tissue section was deparaffinized, rehydrated, and then incubated with fresh 3% hydrogen peroxide in methanol for 15 min. After rinsing with phosphate-buffered saline (PBS), antigen retrieval was carried out by microwave treatment in 0.01 M sodium citrate buffer (pH 6.0) at 100°C for 15 min. Next, non-specific binding was blocked with normal goat serum for 15 min at room temperature, followed by incubation at 4°C overnight with different primary antibodies. Antibodies, clones, dilutions, pretreatment conditions, and sources are listed in Table 2. After rinsing with PBS, slides were incubated with biotin-conjugated secondary antibodies for 10 min at room temperature, followed by incubation with streptavidin-conjugated peroxidase working solution for 10 min. Subsequently, sections were stained for 3-5 min with 3,39-diaminobenzidine tetrahydrochloride (DAB), counterstained with Mayer's hematoxylin, dehydrated, and mounted. Negative controls were prepared by substituting PBS for primary antibody. For this study, the intensity of VEGF and COX-2 staining were scored on a scale of 0-3: 0, negative; 1, light staining; 2, moderate staining; and 3, intense staining. The percentages of positive tumor cells of different intensities (percentage of the surface area covered) were calculated as the number of cells with each intensity score divided by the total number of tumor cells (x 100). Areas that were negative were given a value of 0. A total of 10-12 discrete foci in every section were analyzed to determine average staining intensity and the percentage of the surface area covered. The final histoscore was calculated using the formula: [(1× percentage of weakly positive tumor cells) + (2× percentage of moderately positive tumor cells) + (3× percentage of intensely positive tumor cells)]. The histoscore was estimated independently by two investigators by microscopic examination at 400× magnification. If the histoscores determined by the two investigators differed by more than 15%, a recount was taken to reach agreement. The results of COX-2 and VEGF immunostaining were classified into high and low expression using cut-off values based on the median values of their respective histoscores.

**Table 2:** Multivariate analysis of VEGF and MVD expression in NSCLC specimens

		VEGF expression			MVD expression	
		
	*β*	HR (95% CI)	*P*	*β*	HR (95% CI)	*P*
**COX-2 expression**						
**High**	2.286	9.836 (3.387 - 28.564)	0.000	1.146	3.147 (1.152 - 8.598)	0.025
**Low**	1.000			1.000		
**TNM stage**						
**III + IV**	0.061	1.063 (0.493 - 2.289)	0.877	0.025	1.025 (0.493 - 2.132)	0.947
**I + II**	1.000			1.000		
**Histology**						
**Adeno**	-0.300	0.741 (0.303 - 1.810)	0.510	0.400	1.491 (0.649 - 3.425)	0.346
**SCC**	1.000			1.000		
**Differentiation**						
**Poor**	-0.292	0.746 (0.198 - 2.809)	0.665	-0.969	0.379 (0.106 - 1.359)	0.137
**Well and moderate**	1.000			1.000		
**Smoking**						
**Yes**	-0.775	0.461 (0.145 - 1.461)	0.188	-0.481	0.618 (0.214 - 1.785)	0.374
**No**	1.000			1.000		
**Sex**						
**Male**	-1.005	0.366 (0.101 - 1.330)	0.127	-0.511	0.600 (0.170 - 2.110)	0.426
**Female**	1.000			1.000		
**Age**						
**≥ 60 yrs**	0.316	1.371 (0.413 - 4.551)	0.606	-0.223	0.800 (0.251 - 2.551)	0.706
**< 60 yrs**	1.000			1.000		

Abbreviations: HR, hazard ratio; CI, confidence interval of the estimated HR; Adeno, adenocarcinoma; SCC, squamous cell carcinoma

On the other hand, Immunohistochemical reactions for CD34 antigen were observed independently by two investigators using microscope. The two most vascularized areas within tumor ('hot spots') were chosen at low magnification (×40) and vessels were counted in a representative high magnification (×400; 0.152 mm^2^; 0.44 mm diameter) field in each of these three areas. The high-magnification fields were then marked for subsequent image cytometric analysis. Single immunoreactive endothelial cells, or endothelial cell clusters separating from other microvessels, were counted as individual microvessels. Endothelial staining in large vessels with tunica media and nonspecific staining of non endothelial structures were excluded in microvessel counts. Mean visual microvessel density for CD34 was calculated as the average of six counts (two hot spots and three microscopic fields). The microvessel counts that were higher than the median of the microvessel counts were taken as high MVD, and the microvessel counts that were lower than the median of the microvessel counts were taken as low MVD.

### Measurement of cell viability of NSCLC cells treated with COX-2

Adherent cells in culture flasks were washed three times with serum-free medium, and digested with 0.25% trypsin for 3-5 minutes to dislodge cells from the substrate. Trypsin digestion was stopped by adding medium containing FBS, and a single-cell suspension was obtained by trituration. Cells were seeded at a density of 8 × 10^3 ^cells/well in a 96-well plate, and the space surrounding wells was filled with sterile PBS to prevent dehydration. After incubating for 12 h, cells were treated with COX-2 (diluted 0-3000-fold). After 24 h, 20 μL of a 5-mg/mL MTT solution was added to each well and then cells were cultured for an additional 4 h. The process was terminated by aspirating the medium in each well. After adding 150 μL of dimethyl sulfoxide per well, the plate was agitated by low-speed oscillation for 10 min to allow the crystals to fully dissolve. Absorbance values (OD 490 nm) for each well were measured using an enzyme-linked immunosorbent assay and a Thermo Multiskan Spectrum full-wavelength microplate reader (Thermo Electron Corp., Burlington, ON, Canada). Blank controls (medium) and untreated control cell conditions were included in each assay. Cell viability is expressed as a ratio of the absorbance of treated cells to that of untreated controls. The median effective concentration (EC_50_) for COX-2 was determined by linear regression analysis of the average promotion rate and chemical concentration using EXCEL (version 2003). All experiments were performed three times and the average results were calculated.

### Measurement of VEGF expression in NSCLC cells treated with COX-2

NSCLC cells were carefully washed with a serum-free medium, digested with 0.25% trypsin to generate a single-cell suspension, and then seeded in 6-well plates at 5 × 10^5 ^cells/well. After 12 h of starvation at 37°C and 5% CO_2_, different concentrations of COX-2 were added, and cells were incubated at 37°C and 5% CO_2 _for 12 h. COX-2-treated cells were then digested with 0.25% trypsin to yield a single-cell suspension. The cell suspension was added to two tubes (experimental and control) at 10^8 ^cells/mL, and then fixed by adding 100 μL fixation buffer to each tube and incubating for 15 min. The cells were then washed twice with permeabilization buffer and the supernatant was removed. Mouse anti-human VEGF antibody (1 μL) and human anti-rabbit IgG (1 μL) was added to experimental and control tubes, respectively, and tubes were incubated at room temperature (18°C-25°C) 30 min. After washing cells twice with 500 μL permeabilization buffer, 100 μL fluorescein isothiocyanate (FITC)-conjugated sheep anti-rabbit antibody (diluted 1:200 in permeabilization buffer) was added and tubes were incubated at room temperature for 30 min. Cells were then washed two times with 500 μL permeabilization buffer and 300 μL PBS was added. After preheating a Coulter Elite flow cytometer (Beckman-Coulter Company, Fullerton, CA, USA) for 30 min, correcting the instrument using fluorescent microspheres (laser wavelength, 488 nm) and calibrating using the blank control, 1000 cells were counted and the percentage of positive cells and mean fluorescence intensity were calculated.

### Comparison of VEGF expression in NSCLC cells treated with COX-2 and inhibitors or activators of PKC, PKA, and PGE_2_

Adherent cells in culture flasks were washed three times with serum-free medium, and digested with 0.25% trypsin as described above to obtain a single-cell suspension. Cells were seeded in 6-well plates by adding 1.5 mL of cell suspension (3-5 × 10^5 ^cells/well), and then incubated at 37°C in a humidified 5% CO_2 _atmosphere until reaching confluence. After serum starvation, a suitable concentration of COX-2 was added and cells were incubated for 12 h. Thereafter, AH6809 (50 μM), KT5720 (10 μM), RO-31-8425 (1 μM), or PMA (0.1 μM) was added, as indicated in the text, and cells were incubated for an additional 12 h. Cultures were then trypsin-digested to yield a single-cell suspension and evaluated by flow cytometry to obtain the geometric mean fluorescence intensity of VEGF expression. This experiment was performed three times.

### Statistical analysis

All calculations were done using SPSS v12.0 statistical software (Chicago, IL, USA). Data were presented as mean ± standard deviation. Spearman's coefficient of correlation, Chi-squared tests, and Mann-Whitney tests were used as appropriate. A multivariate model employing logistic regression analysis was used to evaluate the statistical association among variables. For all tests, a two-sided P-value less than 0.05 was considered to be significant. Hazard ratios (HR) and their corresponding 95% confidence intervals (95% CI) were computed to provide quantitative information about the relevance of the results of statistical analyses.

## Results

### Basic clinical information and tumor characteristics

A total of 84 NSCLC patients (63 male and 21 female) treated by curative surgical resection were enrolled in the study; the mean age of the study participants was 58.0 ± 10.3 years (rang, 35-78 years). Of the 84 cases, 34 were lung adenocarcinoma, 45 were squamous cell carcinoma, and five were large-cell carcinoma; 40 cases were well or moderately differentiated and 44 were poorly differentiation. Using the TNM staging system of the International Union Against Cancer (2002) [13], cases were classified as stage I (n = 44), stage II (n = 19), stage III (n = 17), and stage IV (n = 4). Patient data were analyzed after a 5-year follow-up, and information was obtained from 91.6% (77 of 84) of patients. The median overall survival was 26.0 ± 2.4 months; mean overall survival was 39.3 ± 6.2 months.

### COX-2 expression is correlated with VEGF profile in NSCLC tumors

We first observed the association between COX-2 expression and clinicopathologic factors. As shown in Table 1 COX-2 expression varied among tumor samples. Strong COX-2 staining was observed in 45 cases (53.6%), whereas weak staining or no staining was detected in 39 cases (46.4%). COX-2 expression in tumor cells was significantly correlated with MVD (*P *= 0.036) and VEGF expression (*P *= 0.001), but was not correlated with age, sex, smoking, TNM stage, or histology. The strength of the associations between each individual predictor and VEGF or MVD is shown in Table 2. When all of the predictors were included in a multivariate analysis, COX-2 expression in tumor tissue retained a significant association with both VEGF expression and MVD (hazard ratio, 9.836; *P *= 0.001; hazard ratio, 3.147; *P *= 0.025), demonstrating that COX-2 expression in tumor tissue is an independent predictive factor of VEGF expression and MVD in NSCLC patients.

### Effects of COX-2 on tumor-associated VEGF expression

**Figure 1:**
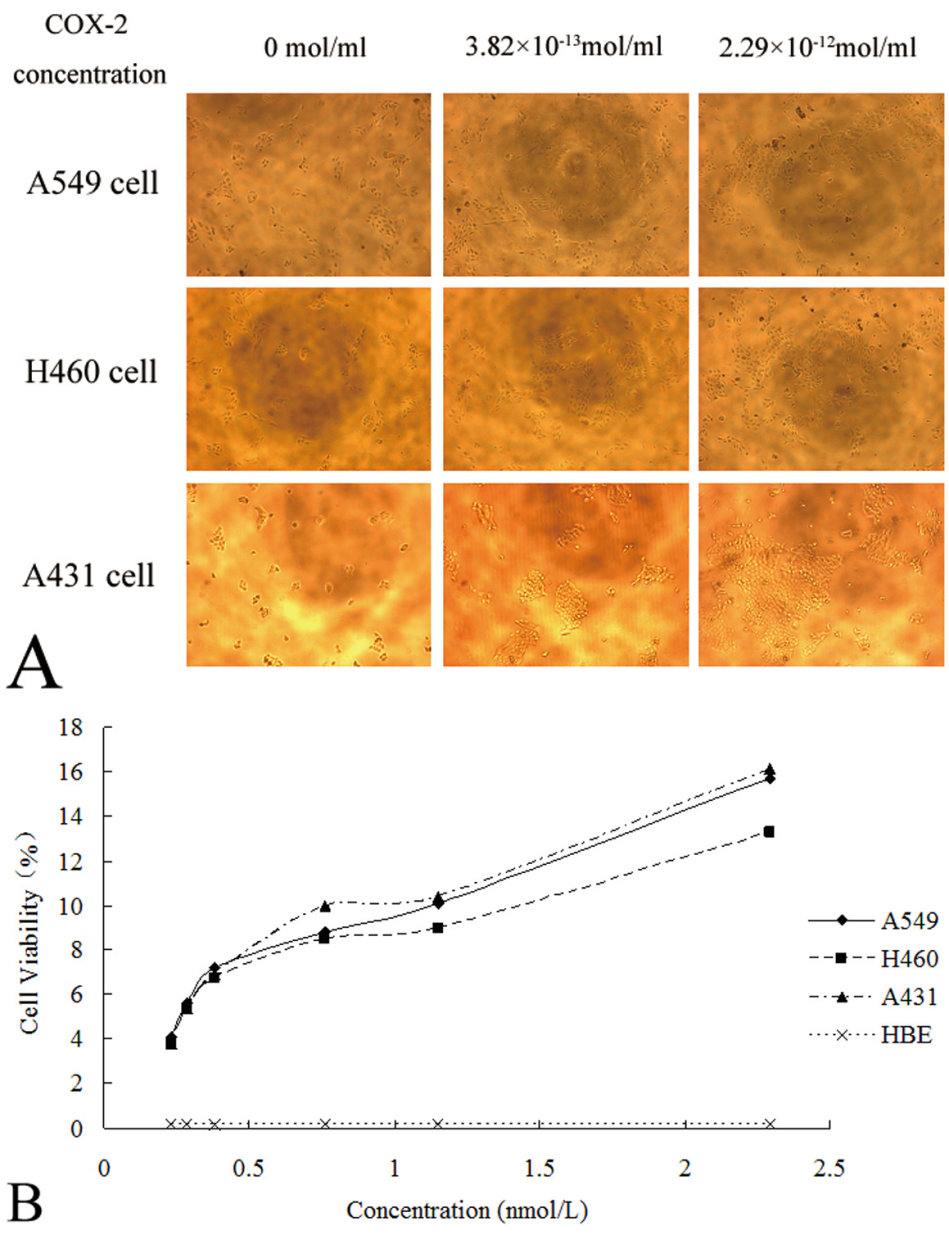
**Cell viability (MTT assay) for determination of EC_50 _of COX-2 stimulation in non-small cell lung cancer cell lines**. **(A**) Prominent increasing in population of A549, H460, and A431 cells were showed in COX-2 concentration of 0, 3.82 × 10^-13^mol/ml, and 2.29 × 10^-12^mol/ml, respectively (×200). (**B**) Curves of cell viability (MTT assay) for determination of EC_50 _in A549 (y = 0.0511× + 0.0424), H460 (y = 0.0408× + 0.043), and A431 cells (y = 0.0543× + 0.0415) were showed. Calculated EC_50 _were 8.95 nmol/L in A549, 11.2 nmol/L in H460, and 8.44 nmol/L in A431 cells.

We next addressed whether COX-2 enhanced the proliferation of NSCLC cells. As demonstrated in Figure 1 treatment with exogenously applied COX-2 induced a prominent dose-dependent increase in the proliferation of the tumor cells used in these assays; in contrast, COX-2 failed to promote the proliferation of HBE cells, used as controls. A linear regression analysis of cell viability showed the EC_50 _values for enhancement of tumor cell growth by COX-2 (concentration required to increase growth by ~50% after a 24-hour treatment) were 8.95 × 10^-3^, 11.20 × 10^-3^, and 8.44 × 10^-3 ^μM for A549, H460 and A431 cells, respectively.

**Figure 2:**
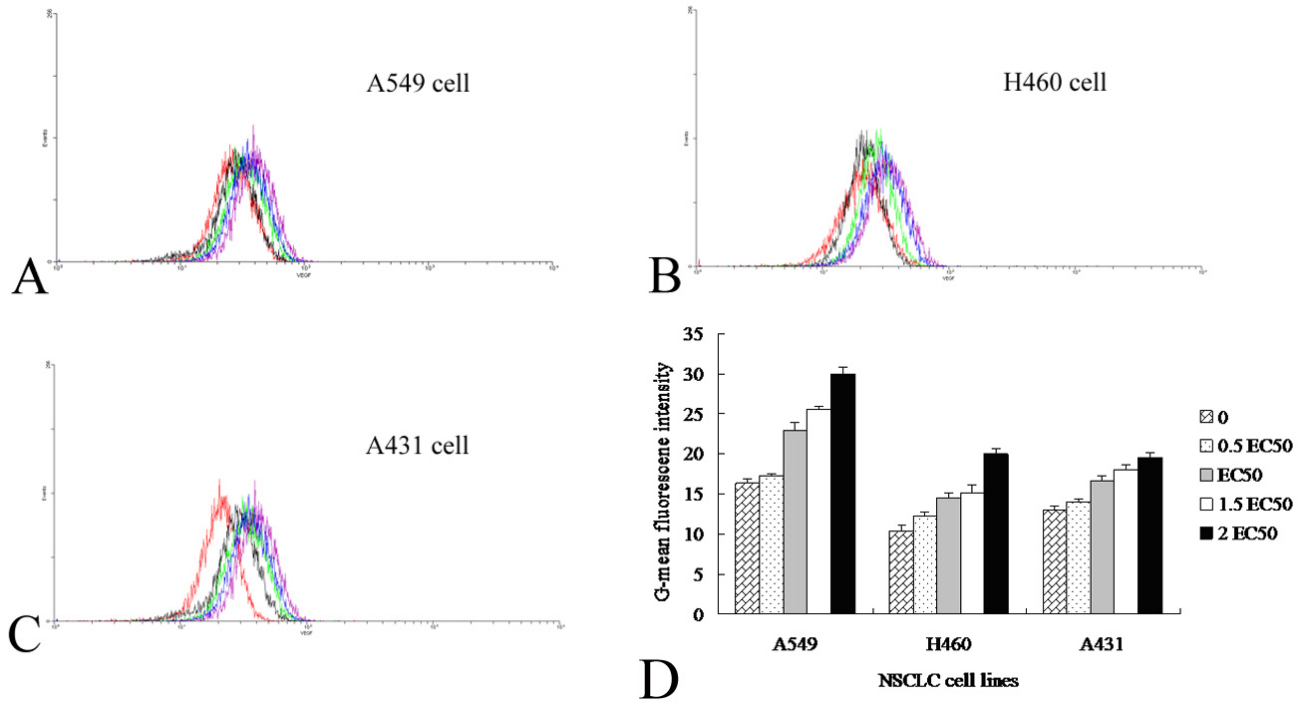
**Determination of the effective concentration for COX-2 mediated VEGF up-regulation in NSCLC cells**. (**A**) In A549 cells, red, purple, green and blue curves represented COX-2 concentrations of 0, 9.17 × 10^-12^mol/ml, 1.83 × 10^-11^mol/ml, and 7.34 × 10^-11^mol/ml, with G-mean fluorescence intensity of 26.32, 32.93, 35.45, and 39.98, respectively. (**B**) In H460 cells, red, purple and green curves represented COX-2 concentrations of 0, 9.17 × 10^-12^mol/ml, 3.67 × 10^-11^mol/ml, with G-mean fluorescence intensity of 25.33, 29.56, and 34.99, respectively. (**C**) In A431 cells, red, purple, green and blue curves represented COX-2 concentrations of 0, 9.17 × 10^-12^mol/ml, 1.83 × 10^-11^mol/ml, and 7.34 × 10^-11^mol/ml, with G-mean fluorescence intensity of 25.98, 33.23, 36.09, and 38.89, respectively. (**D**) COX-2 mediated VEGF up-regulation was shown. G-mean, geometric mean.

We further addressed whether COX-2 enhanced tumor-associated VEGF expression in NSCLC cells, treating tumor cell lines with different concentrations of COX-2 (0.5-, 1-, 1.5-, and 2-times the EC_50 _value). As shown in Figure 2 COX-2 increased the geometric mean fluorescence intensity of VEGF expression in a dose-dependent manner. This phenomenon was especially obvious in A549 and H460 cells. As demonstrated in Figure 1 and 2, the doses of COX-2 that optimally induced VEGF expression without causing a cytotoxic effect were 13.43 × 10^-3^, 16.8 × 10^-3^, and 12.66 × 10^-3 ^μM in A549, H460, and A431 cells, respectively.

### Effect of AH6809, KT5720, and RO-31-8425 on COX-2 stimulation of tumor-associated VEGF expression

**Figure 3:**
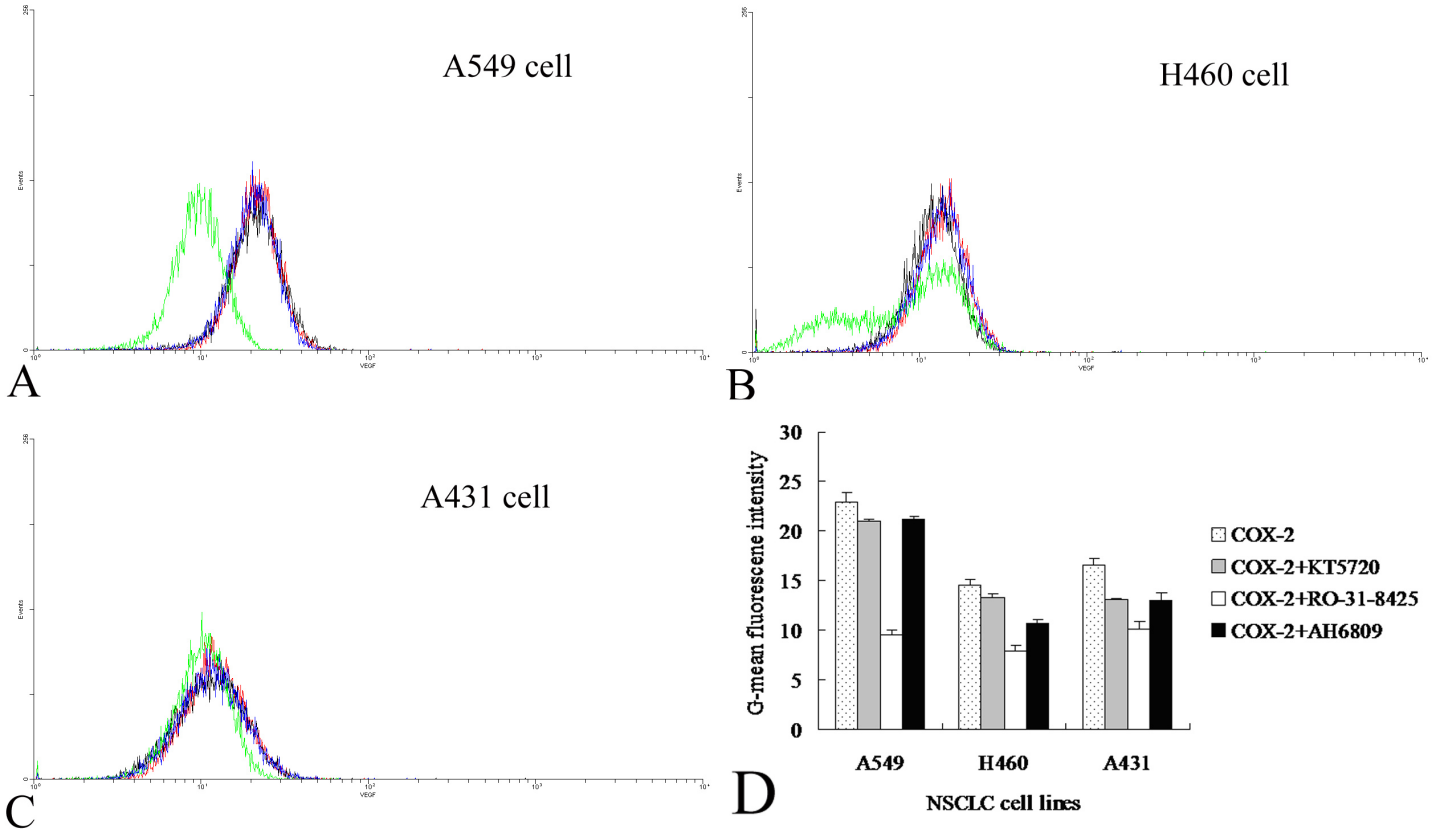
**COX-2 mediated VEGF up-regulation in NSCLC cells was changed with treatment with several reagents**. VEGF expression after treatment with several reagents was showed in A549 (**A**), H460 (**B**), and A431 cells (**C**). Red curve indicated cells treatment with COX-2, black curve indicated with COX-2 and AH6809, green curve indicated with COX-2 and KT5720, and blue curve indicated with COX-2 and RO-31-8425. Comparison of G-mean fluorescence intensity of VEGF was showed (**D**). G-mean, geometric mean.

To explore the mechanism underlying COX-2 involvement in tumor-associated VEGF expression, we employed selective inhibitors of several intracellular signaling pathways. As shown in Figure 3 treatment of NSCLC tumor cells with the PKC inhibitor RO-31-8425 caused a prominent decrease in COX-2-dependent VEGF expression, reducing COX-2-stimulated VEGF expression by 51.1% in A549 cells (*p *< 0.01), 41.2% in H460 cells (*p *< 0.01), and 23.2% in A431 cells (*p *< 0.01) compared with controls. Inhibition of PKA with the selective inhibitor KT5720 did not significantly inhibit COX-2-dependent, tumor-associated VEGF expression in NSCLC cells. Notably, AH680, a selective antagonist of EP1/EP2 receptors, exerted an inhibitory effect on COX-2-dependent VEGF expression in NSCLC cells (*p *< 0.05).

### Effect of PMA on COX-2 stimulation of tumor-associated VEGF expression

**Figure 4:**
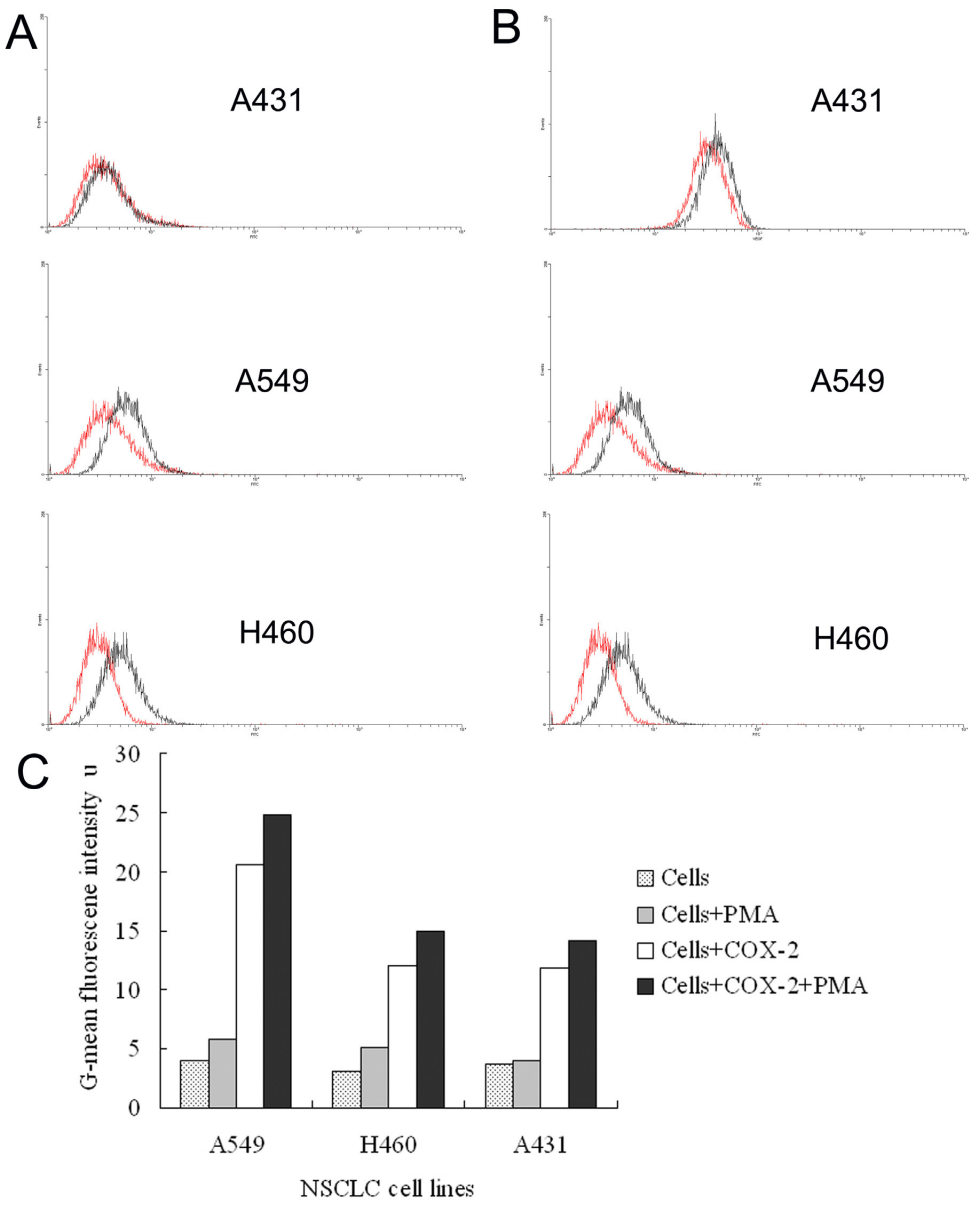
**Effect of COX-2 and PAM on tumor associated VEGF expression in NSCLC cells**. VEGF expression after treatment with PMA was showed in A431, A549, and H460 (**A**). Red curve indicated no treatment, black curve indicated treatment with PMA. VEGF expression after treatment with COX-2 and PMA was showed in A431, A549, and H460 (**B**). Red curve indicated treatment with COX-2, black curve indicated treatment with COX-2 and PMA. Comparison of G-mean fluorescence intensity of VEGF was showed (**C**). G-mean, geometric mean.

To confirm that PKC played a key role in COX-2-dependent, tumor-associated VEGF expression, we treated NSCLC cell lines with the PKC activator PMA. As demonstrated in Figure 4 treatment with both COX-2 and PMA significantly increased the geometric mean fluorescence intensity of VEGF expression in A549, H460, and A431 cells compared to treatment with COX-2 or PMA alone (*p *< 0.01 for all).

## Discussion

Tumor-induced angiogenesis is a cardinal attribute of malignant disease [16]. The microvasculature formed with new blood vessels in tumor stroma mediates transport of nutrients to the tumor cells, and is a prerequisite for growth of tumors beyond a certain size [17]. It is known that malignant angiogenesis is induced by specific angiogenesis-promoting molecules, such as VEGF, which are highly expressed in various types of solid tumors and are released by the tumor itself. The resulting tumor-induced neovasculature exhibits enhanced endothelial cell permeability, and the associated increase in vascular permeability may allow the extravasation of plasma proteins and formation of extracellular matrix favorable to endothelial and stromal cell migration [18]. Importantly, certain molecules, such as COX-2, have been found to participate in up-regulation of VEGF in malignant tissue. COX-2 expression has been implicated in the regulation of VEGF in colonic cancer [19], thyroid cancer [20], and nasopharyngeal carcinoma [21]. Previous studies have demonstrated that COX-2 is able to induce angiogenesis or promote tumor adhesion and metastasis [22,23], and also plays a key role in drug resistance in NSCLC patients [24]. Consistent with this, COX-2 expression has been detected immunohistochemically in NSCLC specimens, including all squamous cell lung cancer and 70% of adenocarcinomas [25]. However, the involvement of COX-2 in the angiogenic response of tumor cells and the role of COX-2 in up-regulating VEGF release by NSCLC cells has been unclear. In order to elucidate the relationship between COX-2 and tumor-associated VEGF expression, we first investigated the association of COX-2 expression in NSCLC tissue samples with clinical and pathologic factors, including VEGF expression and MVD. Our findings indicated a significant difference in VEGF staining and MVD between NSCLC specimens with strong and weak COX-2 expression. When all of the predictors were included in a multivariate analysis, COX-2 expression retained its significant association with VEGF staining and MVD, demonstrating that COX-2 expression is an independent predictive factor for changes in both VEGF expression and MVD in NSCLC tissue. These results suggest that COX-2 may contribute to maintaining a high level of VEGF in NSCLC tissue, thereby playing an important role in tumor-induced angiogenesis.

Previous reports provide no insight into how up-regulating COX-2 might mediate tumor-associated VEGF expression in NSCLC tissue in a physiological context. In order to address this question, we assessed changes in tumor-associated VEGF expression in NSCLC cells that accompany changes in COX-2 by treating cells directly with COX-2 protein. Because this is the first such study, there was no available information on the concentrations of COX-2 that are effective in stimulating proliferation in NSCLC cells in vitro. Accordingly, we used an MTT assay to investigate the characteristic tumor cell responses to COX-2 as a chemical agent in three NSCLC cell lines. Crucially, our data demonstrated that A549, H460, and A431 tumor cells were stimulated to proliferate by exogenously applied COX-2, whereas normal bronchial epithelial cells (HBE) used as a control were not. The EC_50 _values for COX-2 in stimulating proliferation were not substantially different among the tested tumor cell lines. Based on our data, it is reasonable to propose that COX-2 is an active agent in these tested NSCLC cells. We also found using flow cytometry that COX-2 exposure up-regulated tumor-associated VEGF expression in NSCLC cells, exhibiting prominent dose-dependent activity. This phenomenon was particularly evident in A549 lung adenocarcinoma cells. Thus, tumor-associated expression of VEGF may be promoted by COX-2 in NSCLCs.

Although COX-2-mediated VEGF up-regulation in NSCLC has been well studied by several groups [26,27], the detailed molecular mechanism underlying this process had not been previously demonstrated. To explore the linkage between COX-2 and tumor-associated VEGF expression, we employed inhibitors of protein kinase signaling pathways. Our demonstration that COX-2 stimulation of tumor-associated VEGF expression was decreased in NSCLC cells by treatment with selective PKC inhibitors, but not by selective PKA inhibitors, indicates that the contribution of COX-2 to tumor-associated VEGF expression in NSCLC may involve the PKC pathway with no involvement of PKA. This interpretation is supported by results obtained using the PKC activator PMA, which significantly enhanced COX-2-stimulated, tumor-associated VEGF expression without altering VEGF expression when used alone. Thus, the PKC pathway likely plays a role in COX-2-mediated VEGF up-regulation in NSCLC.

Interestingly, our finding that antagonism of the PGE_2 _receptor decreased COX-2-mediated VEGF up-regulation in NSCLC cells, especially in H460 large-cell lung cancer cells, confirms that PGE_2_, a downstream product of COX-2 activity, may participate in COX-2-mediated VEGF up-regulation. Recently, sequential changes in COX-2, downstream PGE_2_, and protein kinase signal transduction pathways have been demonstrated in some tumors [28,29]. PGE_2 _binds to four subtypes of G-protein-coupled receptors--EP1, EP2, EP3, EP4--that activate intracellular signaling cascades. These receptors are distributed on the cell surface and their action depends on PGE_2 _concentration [30]. The EP1 receptor couples to the G_q _subtype and mediates a rise in intracellular calcium concentration; EP2 and EP4 receptors are coupled to the adenylyl cyclase-stimulating G protein G_s_, and mediate a rise in cAMP concentration; by contrast, the EP3 receptor couples to G_i_, inhibiting cyclic AMP generation [31]. Results obtained with AH6809, which inhibits both EP1 and EP2, suggest a G_q_- or G_s_-mediated mechanism, although additional studies will be required to confirm which receptor is the main target on the NSCLC cell surface. Another interesting finding of the present study was the absence of a prominent decrease in COX-2-dependent VEGF activity following inhibition of PGE_2 _receptor(s) in A549 and A431 cells. This result suggests that other prostaglandin components may participate in pathways leading from COX-2 to VEGF expression in different NSCLC cells.

## Conclusions

Our findings demonstrate that COX-2 expression in tumor tissue was an independent predictor of VEGF expression and MVD in NSCLC patients, and COX-2 may be a stimulator of tumor-associated VEGF activity in NSCLC tissue. COX-2-dependent VEGF up-regulation in NSCLC may involve the PKC pathway with no involvement of PKA. Moreover, different downstream prostaglandin products of COX-2 activity may participate in the changes linking COX-2 to VEGF expression in different NSCLC cells.

## Competing interests

The authors declare that they have no competing interests.

## Authors' contributions

The authors contributed to this study as follows: HL, ZC, and HJ conceived of the study; HJ, MZ, SC, LY, JZ, and BZ performed experiments; TW analyzed data and prepared the figures; CZ and HJ drafted the manuscript. All authors have read and approved the final manuscript.
